# Isolation of Aerobic Bacterial Species Associated with Palpable Udder Defects in Non-Dairy Ewes

**DOI:** 10.3390/ani14162317

**Published:** 2024-08-09

**Authors:** Mandefrot M. Zeleke, Paul R. Kenyon, Kate J. Flay, Danielle Aberdein, Sarah J. Pain, Niluka Velathanthiri, Anne L. Ridler

**Affiliations:** 1 School of Veterinary Science, Massey University, Private Bag 11222, Palmerston North 4410, New Zealand; mandemeaza@gmail.com (M.M.Z.); d.aberdein@massey.ac.nz (D.A.); n.velathanthiri@massey.ac.nz (N.V.); 2 School of Agriculture and Environment, Massey University, Private Bag 11222, Palmerston North 4410, New Zealand; p.r.kenyon@massey.ac.nz (P.R.K.); s.j.pain@massey.ac.nz (S.J.P.); 3 Department of Veterinary Clinical Sciences, City University of Hong Kong, Kowloon, Hong Kong 999077, China; kateflay@cityu.edu.hk

**Keywords:** bacteria, non-dairy ewe, hard udder, palpable udder defect, udder lump

## Abstract

**Simple Summary:**

Milk or mammary tissue swab samples were collected from both defective and normal udder halves in three different studies to identify the bacterial species involved. Samples were collected at different physiological time points: pre-mating, throughout lactation, at weaning and post-weaning. Numerous bacterial species were identified, with *Staphylococcus* species, *Mannheimia haemolytica* and Streptococcus being the most frequently isolated. Bacteria were isolated from at least one third (udder lumps) or more than half (hard udder) of defective udder halves, whereas no bacteria were isolated from more than two thirds of normal udder halves. Frequently isolated bacterial species tended to persist longer, whereas less frequently isolated bacterial species showed less stability over time. Bacterial species more frequently identified from defective udder halves and which appear more stable over time should be considered as a factor for making culling decisions.

**Abstract:**

The objectives of these studies were to identify associations between udder half defects (hard or lump) and bacteria isolated from milk or mammary tissue swabs, to compare with samples from normal udder halves at different physiological time points and to compare bacterial species isolated via milk and swabs of mammary tissue from within the same udder halves. A total of 1054 samples were aseptically collected from each udder half of 199 non-dairy breed (Romney) ewes from three different studies (Study A, *n* = 77; Study B, *n* = 74; and Study C, *n* = 48). Conventional bacterial culture and MALDI-ToF mass spectrometry were used for bacterial identification. Of the 225 samples from which bacteria were isolated, *Mannheimia haemolytica* and *Streptococcus uberis* were the dominantly identified species from defective udder halves, whereas coagulase-negative staphylococcus (CNS) species, mostly *Staphylococcus simulans* and *Staphylococcus chromogenes*, were more frequently isolated from normal udder halves. The ongoing presence of bacterial species over time was variable, although less frequently identified species showed less stability over time. A very high agreement (91.5%) of bacterial species identified was observed between the mammary tissue swab and udder half milk samples during post-weaning. In summary, palpable udder half defects were associated with bacterial positivity, and the ongoing presence of the bacteria over time was dependent on the species involved. Hence, culling ewes with palpable udder half defects that had more stable bacterial species could contribute to reducing the recurrence of palpable defects or mastitis.

## 1. Introduction

Udder defects have been associated with reduced milk production and changes in milk composition in both dairy and non-dairy ewes [1,2,3]. Further, low survival rates [4] and reduced live weight gains [5] have been observed in pre-weaned lambs reared by ewes with udder defects. These defects can be associated with mild to clinically noticeable changes in milk production and udder structure [6]. Studies from Canada, the Netherlands, the United Kingdom (UK) and New Zealand have reported that a range of bacterial species have been isolated from either milk or in abscesses from defective udders, with Staphylococci (*S. aureus* and coagulase-negative isolates) and *Mannheimia haemolytica* (*M. haemolytica*) being the most frequently reported isolates [6,7,8,9,10]. These studies also reported isolation of other relatively less frequently isolated bacterial species such as Streptococci, Enterobacteria, Corynebacterium and Pseudomonas. Over the past five decades in New Zealand, a small number of studies investigating bacterial species isolated from defective udders and teats have reported isolation of these and related bacterial species from clinically mastitic milk [7,8,11,12]. These studies were undertaken either shortly after lambing or during the post-weaning period and in most cases involved taking milk samples at a single point in time from ewes whose history of udder defects was unknown.

Upon palpation of an udder, any detectable mass of different consistency from the rest of the gland tissue or diffuse change in consistency of the whole mammary gland can be considered as a palpable udder defect [4]. A diffuse hard consistency of the whole mammary gland is a unilateral or bilateral udder defect with no to a small amount of milk excretion from the gland [8,13]. Authors from New Zealand, UK and Greece have reported that this defect type can be observed during both lactation and the dry or the pregnancy period [4,14,15]. Recently, Ridler et al. [7] reported at least seven different bacterial species from milk samples collected from udder halves categorised as hard, one week after weaning in non-dairy ewes in New Zealand. *Staphylococcus aureus* (*S. aureus*) was identified as the dominant bacterial species in that study, although 19.4% (7/36) of the udder halves categorised as hard yielded no bacteria.

Udder lumps have been described as intramammary masses (IMMs) that can vary in size, consistency and location [4,15]. Smith et al. [10], in the UK, collected mammary gland samples with lumps (abscesses) and reported that *S. aureus* was the most frequently detected bacterial species. Ridler et al. [7], in New Zealand, reported various species of bacteria isolated from milk samples from udder halves that contained lumps, including Staphylococcus, Streptococcus and *Mannheimia*. Distinct subcutaneous lumps immediately cranial or sometimes caudal to the udder have been reported in some ewes at weaning or shortly thereafter [7]. Such lumps were described by Saratsis et al. [15] as bacterial negative cysts filled with milk-like fluid.

Previous studies have shown changes in the percentage of palpable udder defects in repeated examinations within the same flock, indicating the dynamic nature of palpable udder defects [4,14]. Further, Ridler et al. [7] reported changes in udder defect status (defective to normal, defective to other defect category (e.g., hard to lump) or normal to defective) across two visits four to six weeks apart following weaning. A recent study by Zeleke et al. [16] reported that udder half defects, either hard or lump, change over time across different physiological time points. However, there has been no research evaluating bacterial isolation in defective udder halves over time.

Combined, the data indicate that while there has been research on bacterial isolation from udders with palpable udder defects in non-dairy ewes, most were undertaken during the short period between weaning and mating. Moreover, the association between bacterial isolation and udder half defect category or changes in defect category over time has not been studied. Therefore the objectives of the current studies were, firstly, to identify aerobic bacteria species isolated from milk and mammary tissue swab samples collected from normal udder halves and those with palpable defects at different physiological times (pre-mating, early lactation, weaning and post-weaning); secondly, identify associations between udder half defect and bacterial isolation; thirdly, assess changes in bacterial isolation from the same udder halves over time; and lastly, compare bacterial isolation from udder halves sampled via milk samples compared with swabs of mammary tissue.

## 2. Materials and Methods

This manuscript comprises data collected during three separate studies described as studies A, B and C below.

### 2.1. Animal Selection and Management

Study A: Ewes were part of a three-year longitudinal study from 2016 to 2018 at Massey University’s Riverside Farm, Wairarapa, New Zealand. The ewes were managed under standard commercial New Zealand conditions on pasture. A total of 77 Romney ewes aged four to five years were selected based on the following: 1. a history of having had udder half defects in the previous three years (24 ewes); 2. a history of no udder half defects in the previous three years but having an udder half defect identified at weaning on 5 December 2018 (38 ewes); 3. a history of no udder half defects in the previous three years and with no udder half defect at weaning on 5 December 2018 (15 ewes). At weaning on 5 December 2018, milk samples were collected from each udder half for bacterial identification from all 77 ewes.

Study B: Seventy-four Romney ewes that had been culled by the owner due to udder defects were obtained from a single commercial farm in North Canterbury, South Island, New Zealand and were transported to Massey University’s Tuapaka Farm, Palmerston North on 10 May 2019. The ewes remained at Tuapaka Farm for a week under standard commercial pasture grazing conditions. During this time, the udder and teats of each ewe were scored using the system described by Griffiths et al. [4]. Ewes were then slaughtered either at a local abattoir (*n* = 70) or, if deemed unfit for transportation to slaughter, were humanely euthanised at Massey University (*n* = 4). In all cases, the whole udder was collected, transported to Massey University and a mammary gland tissue swab was collected from each udder half within three to six hours (see below for further detail).

Study C: Forty-six ewes joined this study from a three-year (2016 to 2018) longitudinal study at Massey University’s Riverside Farm, Wairarapa, New Zealand based on udder defect history and udder defect status on the selection day. This study comprised lactating ewes with both normal and defective udder halves. Milk samples were collected from each udder half six times during lactation (on approximately days 7, 14, 21, 28, 35 and 42 of lactation), at weaning (29 November 2019) and three weeks after weaning (18 December 2019). Two ewes were culled at day 42 due to routine farm management, and four ewes missed day 42 milk sample collection. Udder halves were scored immediately prior to sample collection on all sampling days. On 21 December 2019, all ewes were slaughtered through a commercial abattoir, and udders were collected for mammary tissue swab sampling (see Section 2.3 below).

### 2.2. Udder Scoring

Each udder half was scored according to Griffiths et al. [4] and categorised into hard, lump or normal. Ewes were restrained and each udder was assessed in a sitting (shearing) position. Initially, each udder half was scored in 7-category score, where scores 1 and 2 describe a “normal” and scores 3 to 6 comprise an udder half with various sizes and consistency of udder lumps. Score 7 was palpated as diffusely hard consistency of the whole udder half. Udder halves categorised as “hard” or “lump” were considered defective.

### 2.3. Post-Mortem Udder Collection

Immediately post-slaughter (Studies B and C), the whole udder was removed, transported to Massey University and washed and thoroughly cleaned.

#### Mammary Swab Sample Collection

A swab sample was collected (Studies B and C) from each udder half (gland) using a sterilized blade to incise the udder skin at the base of teat and at the gland cistern, to expose the whole gland cistern. Several dissections were made using new sterilized blades to check the presence of lumps in the different parts of the udder (cranial or caudal to gland cistern). In each udder half, the gland cistern and/or contents of defects found were swabbed using a transport swab (Fort Richard Laboratories, Auckland, New Zealand). The ewes’ identification number, site of collection, nature of the contents and number of samples were recorded for each udder half independently. The swab samples were cultured immediately or kept for a maximum of 12 h in the refrigerator (4 °C) until culturing.

### 2.4. Milk Sampling

An aseptic milk sampling procedure was conducted to collect milk samples from each udder half (Studies A and C) from live ewes. Before sampling, teats were thoroughly cleaned with 70% ethanol and wiped with commercial teat wipes (Zoetis Mediwipes, Zoetis New Zealand Limited, Auckland, New Zealand). The first three milk squirts were discarded, and then 3–4 mL of milk was collected from each udder half into sterile tubes for bacteriological testing. Milk samples were cultured (see Section 2.5 below) in less than one hour after collection or kept for a maximum of 12 h in the refrigerator (4 °C) until culturing.

### 2.5. Bacterial Culture and Identification

Milk and swab samples were processed at the Massey University School of Veterinary Science bacteriology laboratory. The medium used was Colombia blood agar with 5% sheep blood, which was incubated aerobically at 37 °C for 48 h. Swabs were streaked directly onto the plate. Milk samples (10 µL) were put on a plate using micropipettes and streaked with a sterilized inoculation loop. Cultures with three or more morphologically distinct colonies on a single sample plate were considered positive, and morphological characteristics were assessed [17]. Cultures with three or more different colony types without a dominant colony type were considered contaminated. For all the isolates, single distinct colonies were picked further for sub-culture, Gram stain and catalase and coagulase testing. Purified isolates were then stored at −80 °C until tested using matrix-assisted laser desorption/ionization time-of-flight (MALDI-ToF) mass spectrometry to identify isolates.

### 2.6. Matrix-Assisted Laser Desorption/Ionization Time-of-Flight (MALDI-ToF) Mass Spectrometry Identification

MALDI-ToF mass spectrometry was used to identify isolates [18]. All bacterial isolates were analysed using the direct transfer method, by directly smearing bacterial colony to a target plate (Bruker MALDI Biotyper) at SC Bio Limited, Auckland, New Zealand, and Massey University, Palmerston North, New Zealand. An extended direct method with addition of 1 μL of 70% formic acid or ethanol extraction method was applied for isolates that could not be identified by the direct method. MALDI-TOF results were interpreted based on three categories: high confidence identification (range 2.0–3.0), low confidence identification (range 1.70–1.99) and unidentified (less than 1.70) [19]. Unidentified isolates either at genus or species level were described based on their Gram staining and morphological characteristics.

### 2.7. Statistical Analysis

Chi-squared/Fisher’s test of independency was used to assess associations between udder half defect and bacterial positivity or bacterial species at weaning (Study A), pre-mating (study B) and lactation (days 7, 14, 21, 28, 35 and 42), weaning and post-weaning (Study C). Udder halves with no milk excretion and contaminated culture samples from Study A were excluded from these analyses. However, exclusion of these udder halves did not change the defective to normal proportion difference (*p* > 0.05). The strengths of these associations were measured by Cramer’s V Coefficient [20]. The coefficient ranges from 0 (no association at all) to 1 (perfect association), where high (>0.5), moderate (0.3–0.5), low (0.1–0.3) and little (0–0.1) indicate the range in between.

Multinomial logistic regression was applied to assess the association of time and bacterial positivity using “nnet” package in R statistical software (R-studio 2020 version 4.0) [21]. A lasagna plot was used to visualize longitudinal bacterial positivity (positive/negative or no milk excretion) in an individual udder half across six times in the first six weeks of lactation, at weaning and three weeks post-weaning. In addition, descriptive frequency was used to summarize the ongoing presence of each bacterial species identified in an individual udder half over eight repeated occasions (six times in the first six weeks of lactation, at weaning and three weeks post-weaning). The frequencies were described in three categories: occurrence of only once in an individual udder half, two to three times in an individual udder half or more than four in an individual udder half in those udder halves that excreted milk at least seven times.

The agreement between milk and swab samples from the same udder halves (Study C) was analysed using Cohen’s Kappa [22]. The strength of the Kappa agreement is described as follows: poor (<0.00), slight (0.00–0.20), fair (0.21–0.40), moderate (0.41–0.60), substantial (0.61–0.80) and almost perfect (0.81–1.00).

## 3. Results

### 3.1. Study A: Bacterial Species Identified from Ewes’ Milk Samples Collected at Weaning and Their Association with Udder Defects

In Study A, of the 154 udder halves (from 77 ewes) where attempts were made to collect milk samples, 23 udder halves did not excrete milk (Table 1). No milk was excreted from 3/7 (43%), 11/39 (28%) and 9/108 (8%) of udder halves categorised as hard, lumpy and normal, respectively. Of those that did excrete milk, 41 udder halves were positive for bacteria, 67 had no growth of bacteria and 23 had three or more bacterial colony types with no dominant species and were considered contaminated. All culture-positive udder halves grew a single colony type, except one udder half that grew *Escherichia coli* and *Mannheimia haemolytica*. Bacterial species identified were coagulase-negative staphylococcus (CNS) species, *Mannheimia haemolytica*, *Staphylococcus aureus*, *Streptococcus uberis* and others (Table 1).

Chi-squared testing of independence between bacterial positivity (positive/negative) and udder half defect (hard, lump or normal) was significantly (*p* < 0.05) associated, but the strength of the association was low (Cramer’s V = 0.26, Table 2). In udder halves that excreted milk and with no contamination of bacterial culture, bacteria were isolated from 3/3 (100%) udder halves categorised as hard and 19/25 (76%) udder halves categorised as lump (Table 2). In contrast, 62 out of 81 (76.5%) normal udder halves yielded no bacteria. *M. haemolytica* and Streptococcus species were predominantly isolated from defective udder halves, whereas the majority of CNS species were isolated from normal udder halves. *S. aureus* was isolated from both normal and udder halves categorised as lump. Association between udder half defect and bacterial species was observed, but the strength was weak (*p* = 0.039, Cramer’s V = 0.20, Table 2).

### 3.2. Study B: Bacterial Species Identified from Mammary Swab Samples Collected during Pre-Mating and Their Association with Udder Defects

From a total of 148 udder half (from 74 ewes) mammary swab samples cultured from commercial ewes prior to mating, 91 (61.5%) were culture-negative (Table 1). Eleven udder halves grew three or more dissimilar colony types and were categorised as contaminated. The remaining 46 (31.1%) udder halves were identified as bacterial culture-positive with 43 having a single colony type and three udder halves that yielded two colony types (Table 1). *S. aureus*, *M. haemolytica* and *S. uberis* were the dominant bacterial species identified and were isolated from udder halves categorised as hard, lump and normal (Table 1).

The association between udder half defect and bacterial positivity (positive or negative) was significant (*p* < 0.05), although the strength of the association was weak (Cramer’s V = 0.24; Table 3). Eleven of fourteen (78.6%) udder halves categorised as hard were bacterial culture-positive and one third of the udder halves categorised as lump were culture-positive, whereas most (69.3%) udder halves categorised as normal were culture-negative (Table 3). However, the association between udder half defect and bacterial species was not significant (*p* > 0.05).

As in the case of Study A, *M. haemolytica* and *Streptococcus* species were the major bacterial species isolated from defective udder halves (Table 1). *S. aureus* and *Staphylococcus simulans* (*S. simulans*) were isolated in comparable numbers from both normal and defective udder halves. *Trueperella pyogenes* (*T. pyogenes*) was isolated in lower frequency and only from defective udder halves. A weak association was seen between udder half defect and bacterial species involved (*p* < 0.05, Cramer’s V = 0.24; Table 3).

### 3.3. Study C: Bacterial Species Identified from Milk Samples Collected from Ewes during Lactation, Weaning and Post-Weaning at Keeble Massey University Farm

**Figure 1:**
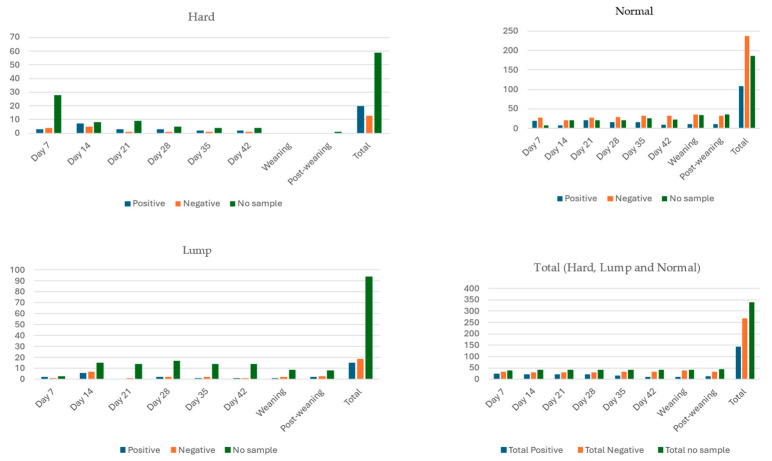
Bacterial positivity of milk samples collected from udder halves categorised as hard, lump or normal from 48 non-dairy breed (Romney) ewes, during the first six weeks of lactation (days 7, 14, 21, 28, 35 and 42), at weaning and three weeks post-weaning (Study C). Note: No data were collected from four ewes on day 42 and two ewes at weaning and three weeks post-weaning.

**Figure 2:**
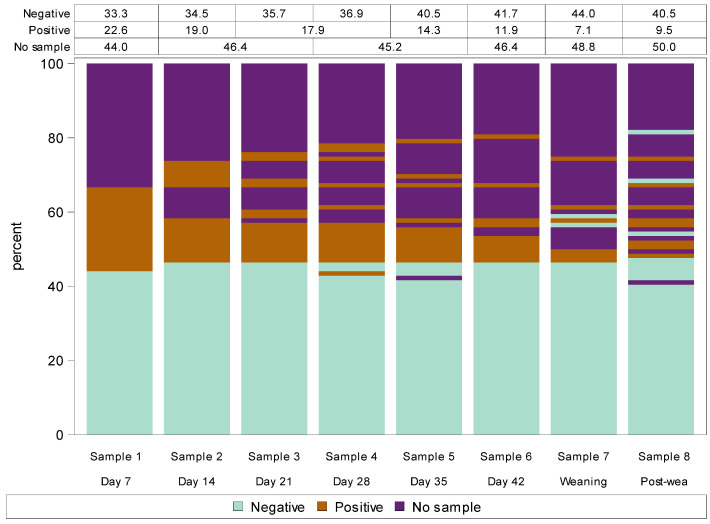
Lasagna plot of bacterial culture positivity (negative, positive or no sample) from eight repeated milk samples collected from each udder half over the first six weeks of lactation (days 7, 14, 21, 28, 35 and 42), at weaning and three weeks post-weaning in 46 non-dairy breed (Romney) ewes (Study C). Note: Each bar shows a different udder half milk collection (i.e., time) while the different colours within each bar represent the bacterial culture result (positive, negative or no sample). The data in the table at the top of the plot designate the percentage of each bacterial culture result at each event, which corresponds to the percentage of each colour at each event. Change in bacterial positivity over time of each udder half can be tracked by following longitudinal transitions across the udder scoring events of stacked bars.

Udder half milk excretion status and bacterial positivity varied throughout six occasions during lactation, at weaning and at post-weaning in 96 udder halves (48 ewes; Figure 1 and Figure 2). The number of udder halves that did not excrete milk varied from 39 on day 7 to 44 at post-weaning. Udder halves categorised as hard, lump and normal were observed on all eight occasions except at weaning when no udder halves were categorised as hard (Figure 1). The frequency of udder halves that were bacterial culture-positive became less frequent as time went on: it was highest at day 7 of lactation and lowest at weaning (Figure 1). From 752 potential udder half milk sampling occurrences, on 339 occasions no sample was able to be obtained. Of the 413 milk samples collected, 5 were contaminated and 270 grew no bacteria, while at least 19 different bacterial species were isolated from the 138 samples that grew bacteria (Table 4). The most commonly isolated bacteria were coagulase-negative staphylococcus (CNS) species, in particular *S. simulans*, *S. chromogenes*, *S. xylosus* and *S. haemolyticus*. *M. haemolytica* and Streptococcus species recorded a reasonably high frequency of occurrence, while the rest of the bacterial species identified were found at low frequency (Table 4).

Multinomial logistic regression analysis of association between udder half bacterial positivity and time (day 7 of lactation through to post-weaning) was only significant (*p* < 0.05) at weaning compared to day 7. The relative risk ratio (±SE) of an udder half being bacterial culture-positive at weaning was 0.39 (±0.45) times compared with day 7 of lactation (the reference).

### 3.4. Study C: Udder Half Bacterial Positivity and Species Identified over Time during Lactation, Weaning and Post-Weaning at Keeble Massey University Farm

Figure 2 describes the repeated culture result from each udder half over the eight milk sampling occasions. The plot demonstrates that udder halves that were bacterial culture-negative on day 7 of lactation almost always remained negative throughout lactation, at weaning and at post-weaning. In the one udder half which did subsequently present a culture-positive result, this only occurred at one sampling time. In contrast, almost all udder halves that were bacterial culture-positive at day 7 either remained positive through to and including post-weaning or subsequently no milk sample was able to be obtained (Figure 2). Most udder halves that did not excrete milk during early lactation continued to not excrete milk until post-weaning; for those that did excrete some milk, it was generally bacterial positive (Figure 2).

To investigate the ongoing presence of bacterial species over time, individual udder halves from which specific bacterial species were isolated were classified into three categories based on how many times they were culture-positive on the eight sampling occasions: single, two to three or more than four occasions. Of the total bacterial species identified, more than six species occurred on only a single occasion in an individual udder half. *Staphylococcus aureus*, *S. uberis* and CNS species such as *S. similans*, *S. chromogenes*, *S. auricularis*, *S. haemolyticus* and *S. warneri* were observed on both one occasion as well as on more than two occasions in an individual udder half. However, *M. haemolytica*, *S. xylosus* and *S. pluranimalium* were only isolated from an individual udder half on more than four occasions, indicating the apparent ongoing presence of these organisms over time (Table A1). Table A1 includes those udder halves that excreted milk on at least six occasions (*n* = 71). *M. haemolytica* (2), *S. chromogenes* (2), *S. aureus* (1), *S. uberis* (1), *S. simulans* (1) and no growth (2) were detected in those udder halves that excreted milk on only one or two occasions (*n* = 9).

### 3.5. Study C: Association of Udder Half Defect and Bacterial Isolation over Lactation, Weaning and Pre-Mating

The association between udder half defect (hard/lump/normal) and bacterial culture positivity (positive/negative) at each time point (days 7, 14, 21, 28, 35 and 42 of lactation, weaning and post-weaning) was not significant (*p* > 0.05). However, *M. haemolytica*, *Streptococcus uberis* and *Staphylococcus aureus* were more frequently isolated (descriptively) from defective udder halves (hard/lump), whereas CNS species were more frequently identified from normal udder halves (Table 5).

### 3.6. Study C: Bacterial Species Identified from Udder Half Mammary Tissue Swab Samples Collected Post-Weaning and Their Association with Udder Half Defect

Swab samples were taken from 92 udder halves (from 46 ewes) at slaughter three weeks after weaning, and only 17 (18%) showed bacterial growth. Four of sixteen udder halves categorised as lump (25%) and 13 of 78 (16.7%) udder halves categorised as normal grew bacteria. There was only one udder half categorised as hard at this time. Fifteen samples grew a single species of bacteria while two udder halves grew two species of bacteria. The bacterial species identified were *Staphylococcus aureus* (2), *S. simulans* (4), *Streptococcus uberis* (2), *Streptococcus pluranimalium* (1), *Acinetobacter indicus* (1), *Trueperella pyogenes* (1), *M. haemolytica* (1), *S. epidermidis* (1), *Staphylococcus xylosus* (1), *Acinetobacter schindlerim* (1) and unidentified Gram-positive rods (2). The association between udder half defect and bacterial positivity was not significant (*p* > 0.05).

From a total of 43 udder halves (nine ewes with both halves and 25 single udder halves) that did not excrete milk on at least six occasions, a culture-positive result was observed from seven udder halves from swab samples post-weaning. No significant (*p* > 0.05) association was observed between udder half defect and bacterial positivity. Swab samples from normal udder halves grew *Staphylococcus aureus*, *S. epidermidis*, *Streptococcus mitis* and *Staphylococcus simulans*, while *Trueperella pyogens* and *Streptococcus uberis* were isolated from lumps.

### 3.7. Study C: Comparison of Bacterial Isolation from Ewes’ Udder Half Milk and Swab Samples Collected at Post-Weaning

From a total of 92 udder halves from which milk sampling was attempted three weeks post-weaning, 46 (50%) did not excrete milk. Thus, the agreement analysis of bacterial isolation from milk and mammary tissue swabs could only be undertaken for the 46 udder halves from which both types of samples were collected. All udder halves that had culture-positive milk samples also had culture-positive swab samples. However, four udder halves with bacterial culture-positive milk samples were bacterial culture-negative from the swab samples. These bacterial species identified from these four milk samples were *S. simulans*, *S.warneri* and *S. capitis*. Udder half swab and milk sampling methods showed a substantial strength of agreement with a k-coefficient of 0.724 (*p* < 0.001).

## 4. Discussion

Overall, more than 30 bacterial species of 10 genera were identified during this study. The type and range of bacteria isolated were similar among populations of sheep and time points of sampling (pre-mating, lactation, weaning and post-weaning). CNS (mainly *S. simulans* and *S. chromogens*), *M. haemolytica*, *Staphylococcus aureus* and *Streptococcus uberis* were the most frequently isolated bacterial species. Previous studies have similarly reported *Staphylococcus aureus*, *M. haemolytica* and CNS as the most frequently isolated bacterial species in non-dairy ewes in New Zealand [7,8,11] and internationally [6,23,24,25].

A significant but weak association between udder half defect and bacterial culture positivity was observed at weaning and pre-mating, whereas no association was found during lactation. Across these three studies, bacteria were isolated from 50–78% of udder halves categorised as hard. Similarly, Ridler et al. [7] isolated bacteria from 28/36 (78%) udder halves categorised as hard. Other authors have also isolated various bacterial species from defective udder halves including those udder halves categorised as hard [8,15]. Bacteria were isolated from 33–61% of udder halves categorised as lump across the three studies. Likewise, in a study undertaken at weaning, 30% of udder halves categorised as lump isolated bacteria [2]. Smith et al. [10] reported identification of several bacterial species from lumps (abscesses), but some failed to grow bacteria. No bacteria were isolated from 69–85% of normal udder halves in the current three studies which is comparable to the 75% previously reported by Ridler et al. [7]. The bacteria isolated from normal udder halves (15–31%) could be due to subclinical mastitis or opportunistic normal skin or environmental microbial flora such as CNS [26]. Marogna et al. [27] analysed the correlation between bacterial positivity and clinical findings such as visible abscesses, nodules and milk abnormalities and reported that ewes with such clinical findings were 105 to 317% more likely to be bacterial culture-positive compared to samples from udders/milk with no abnormalities.

Combining these current studies shows that a considerable percentage of udder halves categorised as either hard or lump isolated no bacteria, while a small percentage of normal udder halves did isolate bacteria. This variation in the association between udder half defect and bacterial positivity might be due to differences in bacterial species pathogenicity (i.e., infection with some species may be more likely to result in palpable defects) or ongoing presence over time with the dynamic nature of the defects [9]. In many cases, the defects were likely to have been chronic and, while they are most likely to have been initiated by bacterial infection, the bacteria may have been cleared from the mammary gland over time. It is possible the negative culture results could also be due to low accuracy of traditional bacterial culture [28]. Overall, the association between palpable udder defects and bacterial culture positivity indicates the effect of aerobic bacteria; however, the weakness of the association could also be suggestive of involvement of pathogens other than aerobic bacteria such as anaerobic bacteria, rickettsial organisms, viruses or sterile cysts [29,30]. Mycoplasma species have been reported to cause contagious agalactia in dairy sheep and goats overseas [31], but this condition has not been reported in sheep in New Zealand, nor have any of the four Mycoplasma species implicated in this condition been isolated from New Zealand sheep. In the present study, Mycoplasma organisms were not tested for.

Among the commonly identified bacterial species in the present studies, *M. haemolytica* and *S. uberis* were predominantly isolated from defective udder halves (hard or lump) during lactation, weaning, post-weaning and pre-mating. Similarly, in previous studies, *M. haemolytica* and *S. uberis* were isolated from hard udder halves or udder halves with lumps in the post-weaning period [7,10]. These bacterial species have also been described as an important and highly frequent bacterial species isolated from clinically mastitic milk in non-dairy breed ewes [6,32,33,34]. Watkins and Jones [35] suggested that *M. haemolytica* was equally important or more significant than *S. aureus*, the latter of which is the most identified bacteria from the udders of dairy ewes [36], possibly due to transmission of the bacteria via lamb suckling. *M. haemolytica* resulted in a higher SCC response than *S. aureus*, which showed the pathogenicity of the pathogen in non-dairy ewes [37]. Experimental infection of ewe udders with *M. haemolytica* has been associated with small nodules with thin-walled abscesses [35] and serious pathological changes with little or no milk excretion in later stages of lactation [38]. *Streptococcus uberis* is a common pathogen found in the milk of both dairy cows and dairy sheep and is known to form mammary gland abscesses in sheep [10,39]. The isolation of this bacterium from udder halves with palpable udder defects in the present study may have been due to the chronicity of the defects, which provided extended time to develop abscesses.

*Staphylococcus aureus* has been reported to be the most commonly identified bacterial species in both dairy and non-dairy ewes with subclinical mastitis [23,24], acute clinical mastitis or chronic mastitic udders with lumps or abscesses [10] and defective udders with lumps, diffuse hardness, nodules or open abscess [15]. In the present study, *S. aureus* was isolated in comparable numbers from defective (lump or hard) and normal udder halves at weaning, pre-mating and isolated predominantly from defective udder halves during lactation. Ridler et al. [7] and Smith et al. [10] isolated the most *S. aureus* from udder halves categorised as hard or lump during the dry period. *S. aureus* have been reported to be less frequent in subclinical infection [6]. Nevertheless, the comparable number of *S. aureus* isolated from udder halves with or without clinically identifiable or palpable udder half defects during the dry period in the present study might be because some of the udder halves categorised as normal in the present studies had a previous history of udder defects.

It has been reported that CNS species are the most prevalent bacteria isolated in clinically normal or subclinical mammary infection [26,36,37]. In the present study, CNS species were predominantly isolated from normal udder halves with no palpable defects, which agrees with [7]. However, CNS species were also isolated from udder halves categorised as either hard or lump, which could suggest potential pathogenicity of some CNS species (such as *S. simulans* and *S. chromogens*) to be associated with clinically noticeable changes, as was observed in experimental infection with *S. simulans* in non-dairy ewes [1]. CNS species have been isolated from up to 20% of clinical mastitis cases in cows and have been referred to as emerging mastitis pathogens [40,41]. *Trueperella pyogenes* was only isolated from swab samples collected at pre-mating and post-weaning from udder halves categorised as hard or lump but not during lactation or from milk samples at weaning. Similarly, this bacterium has been associated with dry period mastitis [15].

Individual udder halves that were bacterial culture-negative at the start of lactation almost always remained negative in repeated milk samples across the first six weeks of lactation, weaning and post-weaning. However, udder halves that were bacterial culture-positive in the first week of lactation almost always either had ongoing presence with positivity or resulted in no excretion of milk at later stages. *M. haemolytica*, *S. xylosus* and *S. pluranimalium* had an ongoing presence observed over four or more weeks in an individual udder half. *S. aureus* and other CNS species were either present for a short period or remained stable for four or more weeks. The other less frequently identified bacterial species mostly occurred for a very short period. This finding agrees with a previous study which reported *M. haemolytica* as being stable in its presence over a three-week period in lactation, but *S. aureus* and CNS were only moderately stable [9]. While udder half defects change over time as described by Zeleke et al. [16] (e.g., defective udder halves changed to normal as days in lactation advanced), the ongoing presence of these commonly identified bacterial species in an udder half may indicate the stability of these bacterial species even after a defective udder half changed to normal. This could cause future defect recurrence in these udder halves or be a potential source of infection for other ewes in the flock.

As expected, those udder halves that did not excrete milk at sample collection in the early weeks of lactation had less chance of excreting milk at later stages of lactation or at post-weaning. However, in those udder halves that did excrete small amounts of milk at later stages, the samples were bacterial culture-positive. In these cases, *M. haemolytica*, *S. chromogenes*, *S. uberis, S. aureus* and *S. simulans* were identified, which may imply the ongoing presence or severity of these species. In those udder halves that did not excrete milk at all during lactation, 83% were bacterial culture-negative in post-mortem swab samples, collected three weeks post-weaning. It is possible that these udder halves were bacterial culture-negative throughout lactation, but it is more likely that they were positive at some time in lactation and then resolved by three weeks post-weaning. In chronic mammary inflammation, the mammary immunity can clear or reduce bacterial load to below limits of detection by bacterial culture at post-weaning [42] and therefore be reported as culture-negative. In those udder halves that did not excrete milk, therefore, assessing the bacterial status while the udder was detected with either diffusely hard or lump during lactation, before changing into normal or another defect category, may provide a satisfactory explanation.

Bacteria identified from mammary swab and milk samples collected in individual ewes post-weaning were almost always (91.3%) in agreement. The only difference occurred in four udder halves that had swab-negative cultures, but the milk sample was positive. The agreement between milk and tissue swab bacterial species identified was higher compared to a previous report by Smith et al. [10]. This might be because most of the lumps in this study were in the gland cistern area and were not capsulated or closed; therefore, if bacteria were present in the lump, they were also present in milk. Alternatively, the three CNS species found only in the milk could be opportunists from the teat skin or environment or possibly transferred to milk by suckling from the mouth of the lamb [42,43]. Nearly all the swab samples collected from palpable udder defects (abscess) in the current studies showed one distinct colony type on culture. Overseas authors have reported that mammary abscesses are often polymicrobial [10,44]. It is possible that not only the presence of abscesses but also the location and degree of capsulation of abscesses may play a role in the bacterial diversity of the abscesses and/or agreement between mammary abscess swab and milk samples. Overall, the very high agreement of bacterial species isolated from milk and mammary tissue swabs indicates that identifying bacterial species in udder halves by milk sampling can be a reliable and practical method.

In the current studies, commonly identified pathogenic bacterial species were associated with udder halves categorised as hard or lump, confirming the most likely cause of these palpable udder half defects. Bacterial species such as *M. haemolytica* were stable over time, which could result in future recurrence of udder defects or act as a source of infection for other ewes. However, in the present study, the number of bacterial species identified to assess ongoing presence over time in an individual udder half was not large enough to undertake statistical analysis; hence, the descriptive analysis should be interpreted with caution. To more accurately describe the bacterial species’ changes over time, a higher number of isolates would be needed for each bacterial species, which would require a greater number of ewes than the current study.

## 5. Conclusions

In conclusion, common bacterial species that were isolated from udder halves categorised as either lump or hard were *M. haemolytica* and *Streptococcus uberis*, whereas CNS species were more commonly isolated from normal udder halves. *S. aureus* was isolated from both defective and normal udder halves in comparable numbers with combined high frequency at pre-mating and weaning but predominantly from defective udder halves during lactation. Udder half milk bacterial positivity was stable in repeated assessment across lactation, weaning and post-weaning; however, the stability over time varied among the species involved. *M. haemolytica, S. xylosus* and *S. pluranimalium* had ongoing presence, while Staphylococcal species including *S. aureus* stability were variable in their ongoing presence. Mammary tissue swab and udder half milk methods of bacteria sampling presented a very high agreement of bacterial positivity and species identified at post-weaning. Hence, identifying ewes with bacterial species predominantly identified from udder halves categorised as hard or lump and which appear more stable over time could contribute to making culling decisions and minimize future recurrence of palpable defects or mastitis. Future research is required to further explore the role of specific bacterial species and other pathogens and their relationship to udder defects and changes over time.

## Figures and Tables

**Table 1:** Frequency of bacterial species identified from milk samples collected at weaning (Study A, *n* = 77 ewes) and swab samples collected at pre-mating (Study B; *n* = 74 ewes) from udder halves categorised as hard, lump and normal in non-dairy breed (Romney) ewes.

Bacterial Species	Study A (Weaning, 2018)				Study B (Pre-Mating, 2019)			
	Hard	Lump	Normal	Study A Total	Hard	Lump	Normal	Study B Total
*Staphylococcus aureus*	0	3	3	6	2	1	3	6
*Staphylococcus caprae*	0	0	2	2				
*Staphylococcus chromogenes*	0	1	3	4	0	0	1	1
*Staphylococcus simulans*	1	2	3	6	0	3	3	6
*Staphylococcus devriesei*	0	0	1	1				
*Staphylococcus warnerii*	0	0	3	3				
CNS (species unidentified)	0	1	0	1	0	1	0	1
*Streptococcus oralis*	0	1	0	1				
*Streptococcus pluranimalium*	0	0	1	1				
*Streptococcus uberis*	0	3	1	4	1	3	1	5
*Streptococcus salivarius*					0	1	0	1
*Streptococcus suis*					0	2		2
*Streptococcus* (species unidentified)	0	3	0	3	0	0	1	1
*Mannheimia haemolytica*	1	5	1	7	4	0	2	6
*Arcanobacterium pluranimalium*	1	0	0	1	1	0	0	1
*Trueperella pyogenes*					2	1	0	3
*Corynebacterium* *pseudotuberculosis*					0	1	1	2
*Helcococcus ovis*					0	1	0	1
Gram-positive bacilli					0	0	2	2
Mixed infection								
*Escherichia coli* and *M. haemolytica*	0	0	1	1	1	2	0	3
*Staphylococcus aureus* and *Helcococcus ovis*					0	1	0	1
*S. parasanguinis* and *Neisseria flavescens*					0	0	1	1
*S. uberis* and *Helcococcus ovis*					0	1	0	1
*S. uberis* and *Streptococcus suis*					0	1	0	1
*Streptococcus* spp. and *Staphylococcus* spp.					0	0	1	1
Contamination *	1	3	19	23	0	5	6	11
No bacterial growth	0	6	61	67	3	34	54	91
No milk sample excreted	3	11	9	23				
TOTAL	7	39	108	154	14	58	76	148

* Bacterial cultures with three or more dissimilar colony types. CNS: coagulase-negative staphylococcus. Note: Culture of samples from most udder halves presented a single colony type, but one udder half from Study A and three udder halves from Study B each yielded two distinct types of colonies.

**Table 2:** Association between udder half defects (hard, lump or normal) and bacteria identified from 108 udder half milk samples collected at weaning (Study A, 5 December 2018) from 77 non-dairy breed (Romney) ewes.

	Udder Half Defects					
Parameters	Hard	Lump	Normal	Total	X^2^ (*p*-Value)	Cramer’s V
Bacterial positivity						
Positive	3	19	19	41	<0.05	0.26
Negative	0	6	62	68		
Bacterial species						
*M. haemolytica*	1	5	1	7	<0.05	0.23
*S. aureus*	0	3	3	6		
*Streptococcus*	0	7	2	9		
CNS	1	4	12	17		
Others	1	0	2	3		

CNS: coagulase-negative staphylococcus. Note: Twenty-three udder halves excreted no milk and 23 udder halves with three or more dissimilar colony types (contaminated) were excluded from the analysis.

**Table 3:** Association between udder half defect (hard, lump or normal) and bacteria identified from 148 udder half swabs of mammary tissue from 74 non-dairy breed (Romney) ewes at pre-mating (Study B, 10 May 2019).

	Udder Half Defect Category					
Parameters	Hard	Lump	Normal	Total	X^2^ (*p*-Value)	Cramer’s V
Bacterial positivity						
Positive	11	20	15	46	>0.05	0.24
Negative	3	34	54	91		
Bacterial species						
*M. haemolytica*	4		2	6	<0.05	0.25
*S. aureus*	2	1	3	6		
*Streptococcus*	1	7	1	9		
CNS		4	4	8		
Mixed infection		4	2	6		
Gram-positive bacilli	4	4	3	11		

CNS: coagulase-negative staphylococcus. Note: Eleven udder halves with three or more dissimilar colony types (contaminated) were excluded from the analysis.

**Table 4:** Bacterial species identified from milk samples collected from non-dairy breed (Romney) ewes in the first six weeks of lactation (days 7, 14, 21, 28, 35 and 42), at weaning and three weeks post-weaning (Study C).

Bacterial Spp.	Day 7	Day 14	Day 21	Day 28	Day 35	Day 42	Weaning	Post-Weaning	Total
*Aerococcus viridans*				1					1
*Corynebacterium lipophiloflavum*	1	2	1						4
*Escherichia coli*			1						1
*Kocuria carniphila*				1		1			2
*Mannheimia haemolytica*	3	1			1	1	1	1	8
*Staphylococcus aureus*	1	2	1			1			5
*Staphylococcus auricularis*	2	1	1		1	1			6
*Staphylococcus capitis*				2				1	3
*Staphylococcus chromogenes*		2	3	2	3	1		2	13
*Staphylococcus haemolyticus*	1	1	1	1	1	1	3		9
*Staphylococcus petrasii*			1			1			2
*Staphylococcus simulans*	5	5	5	5	5	2	3	4	34
*Staphylococcus warneri*			1	1	1		1	1	5
*Staphylococcus xylosus*	2	2	2	2	2		1	1	12
*Streptococcus pluranimalium*	1	1	1	1	1	1		1	7
*Streptococcus uberis*	1	2	2	4	2	1	1	1	14
*Staphylococcus auricularis* and *Streptococcus parasanguinis*							1		1
*Aerococcus viridans* and *Staphylococcus simulans*	1								1
*Corynebacterium* spp.		1							1
*Psychrobacter* spp.			1						1
Gram-positive bacilli	1		1					1	3
CNS-unidentified		1	1	1	1	1			5
Contaminated *	5								5
No bacterial growth	33	32	30	32	35	35	38	35	270
No Sample	39	43	43	43	43	41	43	44	339
Total	96	96	96	96	96	88	92	92	752

CNS: Coagulase-negative staphylococcus. * Bacterial cultures with three or more dissimilar colony types.

**Table 5:** Frequency of bacterial species identified from udder halves categorised as hard, lump or normal from 46 non-dairy breed (Romney) ewes, during the first six weeks of lactation, at weaning and three weeks post-weaning (Study C).

Bacterial Species	Udder Half Defect Category			
	Hard	Lump	Normal	Total
*Staphylococcus aureus*	3	1	1	5
*Staphylococcus capitis*			3	3
*Staphylococcus chromogenes*			13	13
*Staphylococcus haemolyticus*			9	9
*Staphylococcus petrasii*			2	2
*Staphylococcus simulans*	4	3	27	34
*Staphylococcus warneri*	1		4	5
*Staphylococcus xylosus*	1	1	10	12
*Staphylococcus auricularis*	1		5	6
*Streptococcus pluranimalium*		2	5	7
*Streptococcus uberis*	6	1	7	14
*Mannheimia haemolytica*	2	5	1	8
*Aerococcus viridans*			1	1
*Corynebacterium lipophiloflavum*	1		3	4
*Escherichia coli*			1	1
*Kocuria carniphila*			2	2
*Corynebacterium* spp.	1			1
*Aerococcus viridans* and *Staphylococcus simulans*			1	1
*Staphylococcus auricularis* and *Streptococcus parasanguinis*			1	1
*Psychrobacter* spp.			1	1
CNS (unidentified)			5	5
Gram-positive bacilli		1	2	3
Contaminated *		1	4	5
No bacterial growth	13	19	238	270
No milk excreted	59	94	186	339
Total	92	128	532	752

* Bacterial cultures with three or more dissimilar colony types.

## Data Availability

The data utilised by this study are available upon request from the corresponding author.

## Appendix A

**Table A1:** Frequency of isolation of bacterial species from milk samples collected from individual udder halves on eight occasions during the first six weeks of lactation (days 7, 14, 21, 28, 35 and 42), at weaning and three weeks post-weaning, from 40 non-dairy breed (Romney) ewes (Study C).

Bacterial Species	Number of Individual Udder Halves		
	Single Occasion	Two or Three Occasions	Four or More Occasions
*A. viridans*	1		
*C. lipophiloflavum*	4		
*E. coli*	1		
*K. carniphila*	2		
*S. petrasii*	2		
*Psychrobacter* spp.	1		
*A. viridans* and *S. simulans*	1		
*S. auricularis* and *S. parasanguinis*	1		
Contaminated	4		
Gram-positive bacilli	1		
*S. aureus*	1	1	
*S. capitis*		1	
CNS-unidentified	3	1	
*S. simulans*	2		6
*S. warneri*	1		1
*S. haemolyticus*	1		1
*S. auricularis*	1		1
*S. chromogenes*	2	1	1
*S. uberis*	1	1	2
*M. haemolytica*			1
*S. xylosus*			2
*S. pluranimalium*			1

Note that this table only includes data from udder halves where milk samples were collected on six or more occasions.
